# Discrimination Contours for Moving Sounds Reveal Duration and Distance Cues Dominate Auditory Speed Perception

**DOI:** 10.1371/journal.pone.0102864

**Published:** 2014-07-30

**Authors:** Tom C. A. Freeman, Johahn Leung, Ella Wufong, Emily Orchard-Mills, Simon Carlile, David Alais

**Affiliations:** 1 School of Psychology, Cardiff University, Cardiff, United Kingdom; 2 Auditory Neuroscience Laboratory, Department of Physiology and Bosch Institute, School of Medicine, University of Sydney, Sydney, New South Wales, Australia; 3 School of Psychology, University of Sydney, Sydney, New South Wales, Australia; Human Brain Research Center, Japan

## Abstract

Evidence that the auditory system contains specialised motion detectors is mixed. Many psychophysical studies confound speed cues with distance and duration cues and present sound sources that do not appear to move in external space. Here we use the ‘discrimination contours’ technique to probe the probabilistic combination of speed, distance and duration for stimuli moving in a horizontal arc around the listener in virtual auditory space. The technique produces a set of motion discrimination thresholds that define a contour in the distance-duration plane for different combination of the three cues, based on a 3-interval oddity task. The orientation of the contour (typically elliptical in shape) reveals which cue or combination of cues dominates. If the auditory system contains specialised motion detectors, stimuli moving over different distances and durations but defining the same speed should be more difficult to discriminate. The resulting discrimination contours should therefore be oriented obliquely along iso-speed lines within the distance-duration plane. However, we found that over a wide range of speeds, distances and durations, the ellipses aligned with distance-duration axes and were stretched vertically, suggesting that listeners were most sensitive to duration. A second experiment showed that listeners were able to make speed judgements when distance and duration cues were degraded by noise, but that performance was worse. Our results therefore suggest that speed is not a primary cue to motion in the auditory system, but that listeners are able to use speed to make discrimination judgements when distance and duration cues are unreliable.

## Introduction

In everyday listening it is rare for location cues to remain stationary over time since the head is often in motion and many sound sources also move. Perceiving auditory space therefore depends on the ability to encode motion within the acoustic image. Various cortical areas are sensitive to the movement of sounds [1]–[5], with the right hemisphere appearing to dominate [6]–[8]. Less clear is the type of motion processing that occurs within these areas. Motion could be recovered directly as a velocity code from dynamic changes within the acoustic image, such as the temporal derivative of interaural level differences (ILDs) [9] or interaural time differences (ITDs). According to this type of account, listeners should be quite sensitive to speed within the acoustic image and the auditory system should contain specialised mechanisms that encode image motion, perhaps similar to the motion detectors found early in the visual system [10], [11]. There is some neurophysiological evidence for this kind of detector, although the evidence for wide-ranging speed and directional selectivity is not strong [12]–[18]. Nonetheless, if such a scheme were to operate then speed should dominate the detection and discrimination of moving sounds. Alternatively, motion could be recovered indirectly by monitoring the locations of sounds at different times and inferring movement as a change in position over time, as in the ‘snapshot theory’ [19]–[21]. Accordingly, listeners should be less reliant on speed *per se*, instead basing their psychophysical judgements on the overall duration shown and distance travelled.

The evidence attempting to differentiate between these two auditory motion accounts is somewhat inconclusive. The existence of a compelling motion aftereffect (MAE) following adaptation to auditory motion would provide simple support for the presence of specialised motion mechanisms, because ‘If you can adapt it, it's there’ (p.479) [22]. However, while adaptation to auditory motion can produce a MAE [8], [23], [24], the effect is somewhat weak [25] compared to the robust MAEs reported for vision [26]. A second line of attack has been to compare the ability to discriminate position when sounds are either static or moving, the idea being that better performance with moving sounds would reveal the existence of specialised motion detectors. Results from these studies are mixed, with some showing that discrimination thresholds for moving stimuli are never better than those found for static stimuli [19], [27]–[29], while others show better performance when stimuli move, at least for slower velocities around 20°/s [20], [30]. In any case, it is unclear whether the existence of specialised motion detectors should necessarily yield better performance when sounds move. This would depend in part on the underlying noise associated with the putative motion mechanism, compared to that related to the processing of duration and distance, and whether observers are able to monitor all three types of information at once.

A more fundamental problem with these studies is that the thresholds they measure necessarily confound speed with duration and distance [27]. This bears a striking analogy to some of the problems encountered in similar experiments in vision (see [31] for review). Moreover, many of the experiments in audition create motion on the basis of isolated cues (e.g., dynamic changes in ITDs or ILDs), potentially creating conflicting information as to the true motion of the stimulus while at the same time reducing ecological validity. In order to circumvent these issues, Carlile & Best (2002)[32] used virtual auditory space (VAS) techniques to present all available motion cues to the listener, including any consequent spectral changes, and also had listeners make speed discrimination judgements with stimulus duration randomised (i.e., roved). The latter is a methodological trick often used in psychophysical studies of visual motion perception (e.g., [33]), one that forces observers to use speed to make their judgements as opposed to distance travelled or total duration. Under this regime, Carlile and Best (2002) found that listeners were able to discriminate motion on the basis of speed alone, but that thresholds improved when cues to distance, duration, start-points and end-points were introduced.

The findings of Carlile & Best (2002) show that listeners are able to use speed to discriminate moving sounds when forced, but they still do not tell us whether auditory motion is a directly-sensed perceptual dimension or one inferred from snapshot-like mechanisms. That is, their data do not speak to the presence or absence of specialised motion detectors in the auditory system. For instance, the improved thresholds obtained when more cues to motion are added could be statistical, arising from probability summation rather than indicating the presence of specialised auditory motion mechanisms.

One useful technique for determining how different motion cues are integrated is to determine discrimination contours for stimuli lying in the distance–duration plane. This approach has been used mainly in colour vision [34]–[37] although more recently it has been applied to visual motion perception [38]–[40] and its potential for studying auditory motion was hinted at by Middlebrooks & Green (1991) in their review on sound localisation [41]. In this paper, we report the first use of the ‘discrimination contours’ technique to investigate the probabilistic combination of distance, duration and speed cues in auditory motion perception. In Experiment 1 we measured discrimination contours for a broadband noise stimulus that moved over a wide range of standard speeds (12.5–200°/s), durations (200–800 ms) and distances (10–40°). Stimuli were presented using VAS to minimise conflicting cues to motion (e.g., changes in ITD with no consequent ILD change) and were individualised for each participant to ensure an externalised motion percept. We found good evidence that duration and to a lesser extent distance dominated the speed cue at threshold. In Experiment 2 we therefore added random components to duration and distance to make them uninformative and found that listeners can use speed alone to discriminate motion when the component cues are unreliable. Our results reveal that auditory motion perception is predominantly driven by distance and duration cues but that speed-based perception is possible when these cues are made uninformative.

## Experiment 1

**Figure 1:**
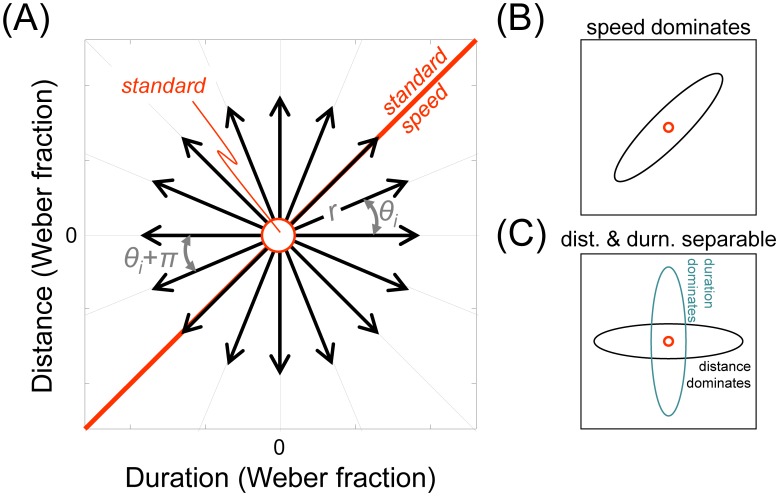
The ‘discrimination contours’ technique. (A) Motion discrimination contours were defined in the distance-duration plane by measuring thresholds along orientations θ_i_ using an 3-interval oddity task consisting of two identical standard stimuli and one test stimulus, presented in a random order. The test differed from the standards by a given proportion (Weber fraction) of duration and distance; a test with an identical speed to the standards therefore falls anywhere on the thick red ‘iso-speed’ line oriented at θ = 45°. (B) If speed dominates performance, then the ellipse will be oriented obliquely along the iso-speed line θ = 45°. (C) If distance and duration cues are separable and dominate performance, then the resulting motion discrimination contours will be aligned with the cardinal axes and tend to be elliptical. When the major axis is horizontal, distance cues dominate; when the major axis vertical, duration cues dominate.

The ‘discrimination contours’ technique is sketched in Figure 1. Discrimination thresholds are determined in a number of different orientations (θ) from a standard stimulus in the distance–duration plane. An oddity task is used to measure each threshold, which consists of presenting listeners with three intervals on each trial and asking them to choose which stimulus is unique. Two of the intervals contain an identical ‘standard’ stimulus while the other interval contains a ‘test’ stimulus, differing in a way that depends on the particular θ being tested. One advantage of the oddity task is that observers are not directed to use a particular cue, such as being told to use speed to find the faster interval as in Carlile & Best (2002). As such, they may use any cue that optimises their performance (though we emphasise that no feedback is given from trial to trial). Because distance and duration cues have different units, we express all stimuli as a proportion of the standard's distance and duration, i.e., as Weber fractions. In standardised Weber units, all stimuli moving at the same speed must lie on θ = 45°, shown as a thick red ‘iso-speed’ line in Figure 1, even if they are composed of different distance–duration combinations. Points lying anywhere else in the distance–duration plane will differ in speed from the standard (and potentially distance and duration cues, depending on the particular θ). Thus, if auditory motion were encoded by specialised detectors sensitive to auditory speed, relatively small speed deviations from the standard speed would be discriminable along lines oriented away from θ = 45°. In contrast, the listener would find discrimination along the iso-speed line particularly difficult compared to discriminations that lie orthogonal to this, where speed changes maximally. We would then expect the subsequent discrimination contour to be an ellipse oriented along the oblique as shown in Figure 1B. On the other hand, if distance and duration cues are separable at threshold and dominate performance, then the contours would be oriented parallel to the cardinal axes as shown in Figure 1C. The major axis of the ellipse will be vertical if sensitivity to duration is better than distance, or horizontal if sensitivity to distance is better.

### Methods

#### Stimuli

A moving broadband (300 Hz–16 kHz) white noise was used as the auditory stimulus, which was rendered using individualised VAS and delivered via Etymotics ER2 insert earphones. The stimuli were driven by an RME Fireface 400 audio interface at a sampling rate of 48 kHz. Details on how individualised VAS is created are described more fully in Pralong & Carlile (1996)[42]. Briefly, we first recorded each listener's head-related transfer functions (HRTFs) at 1° intervals in a 360° arc on the azimuthal plane. To create the moving stimuli, sequential segments of broadband white noise were filtered with the HRTFs corresponding to closely-spaced locations along the intended path of movement. These sequential segments were smoothly concatenated by joining the final conditions of the current filtering process with the initial conditions of the next, using 10 ms raised cosine ramps and an ‘overlap-and-add’ blending method. The HRTF recordings were performed in an anechoic chamber of size 64 m^3^ that was equipped with a computer-controlled, laser-calibrated, robotic hoop (radius 1 m), with a speaker (Audience A3 wideband tweeter) mounted at the apex. Listeners were seated in the center of the room and monitored by an Intersense IC3 magnetic headtracker. At each location, a series of exponential sine sweeps were played [43] and the HRTFs were recorded from insert microphones placed in the subjects ears (Knowles FG2335, 2.8 mm diameter), based on a “blocked-ear” recording technique (see Middlebrooks et al, 1989; Moller et al, 1995).

#### Psychophysical Procedure

**Figure 2:**
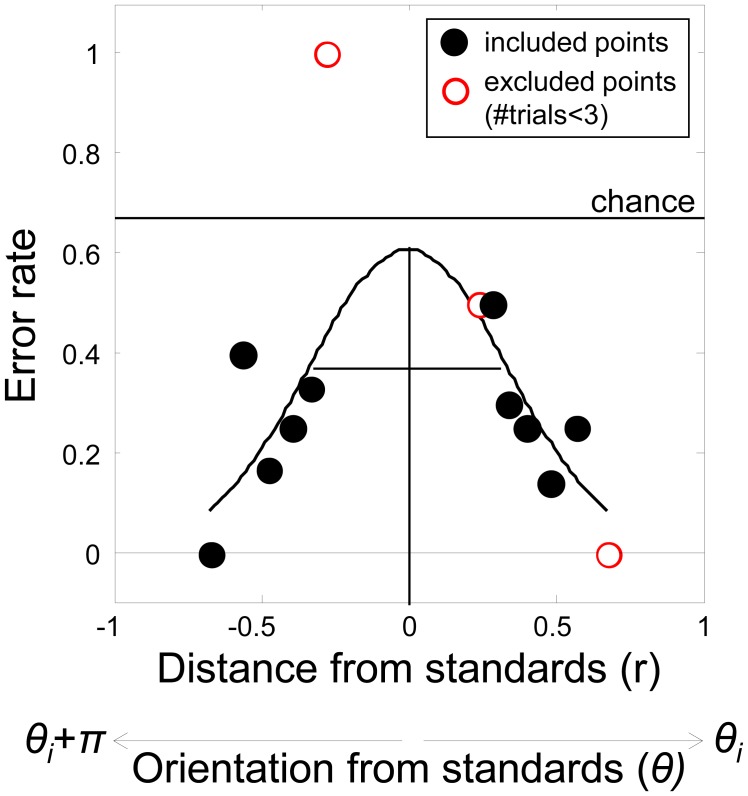
Example psychometric function for single observer. Performance in an 3-interval oddity task follows a Gaussian when error rate is plotted against the test's radial distance (r) along a given orientation θ_i_ and its complement θ_i_+π. Gaussian functions were fit to the data using a maximum likelihood procedure. Any radial test distance containing two or fewer trials was excluded from the fit (examples shown in open red symbols).

Thresholds were determined for a set of orientations (θ_i_) in the distance–duration plane using an oddity task. Each trial consisted of three sequentially-presented intervals, two standards (S) and one test (T), presented in a random order. The listener's task was to pick the odd one out by indicating which stimulus appeared most different using a button press. No feedback was given. The mid-point of each stimulus arc was jittered independently by ±5% of the standard distance in order to make position cues at the start and end of the motion sequences uninformative. The test differed from the standards by given proportions (Weber fractions, W) of the standard duration and distance. Specifically, W_x_ = (T_x_-S_x_)/S_x_ and W_t_ = (T_t_-S_t_)/S_t_, where x refers to distance travelled and t the stimulus duration. Hence, in polar coordinates, the test differed from the standard by a radial distance r = √(W_x_^2^+W_t_^2^) along any θ_i_, as shown in Figure 1A. When r = 0, test and standard are equal, and listeners will be at chance in their ability to correctly identify the test. Hence the error rate = 0.66. As r increases along θ_i_ (or its complement θ_i_+π), error rate declines and describes a Gaussian-shaped psychometric function (Figure 2)[44]. We defined threshold as the standard deviation of the best-fitting Gaussian (see below).

In each experimental session, two θs and their complements were selected at random from the 16 orientations investigated in total. The radial distance r that was used to define the test on any particular trial was controlled by a 1-up 2-down staircase. Each θ was probed with its own staircase, hence each session comprised 4 interleaved staircases. Staircases terminated after 8 reversals.

Nine standard stimuli were constructed from a factorial combination of 3 standard durations (200, 400, 800 ms) and 3 standard distances (10, 20 and 40 degs). This yielded 5 standard speeds (12.5, 25, 50, 100 and 200°/s). Two listeners (L1, L2) generated discrimination contours for each of the 9 standards and two further listeners (L3, L4) completed a subset (3 standard speeds of 12.5, 50 and 200°/s). Three of the listeners (L2-L4) completed two replications for each of the 16 staircases associated with each standard while one listener (L1) completed at least one replication.

#### Analysis

The staircase data along θ_i_ and its complement θ_i_+π were used to construct psychometric functions, an example of which is shown in Figure 2. A maximum-likelihood procedure was used to find the best-fitting Gaussian. The fitting procedure ignored any test stimulus values (r) that had 3 or fewer trials (examples of excluded data points are shown as open red symbols in the Figure). The model included a lapse rate parameter constrained to be 6% or less [45]. Confidence intervals (95%) were estimated from a bootstrapping procedure based on 999 resamples of the data (with replacement) associated with each psychometric function. The bootstrapped means were sorted and the values enclosing the central 95% of the distribution defined the confidence intervals. The bootstrapping distribution was asymmetric; hence the error bars shown in the Results section are as well.

Discrimination contours were summarised by fitting ellipses to the set of thresholds associated with each standard. The best-fitting ellipse was based on an iterative technique that minimises the geometric distance between data and curve (see [38]).

#### Participants

Four listeners took part in the two experiments. Three were authors (L2-L4), two of whom were fully aware of the hypotheses (L2, L3). The other listener (L1) was naïve to the aims of the study. All subjects had normal hearing according to standard clinical audiometry exams.

#### Ethics Statement

Participants gave written informed consent. The experimental procedure conformed to the declaration of Helsinki and was approved by the local ethics committee (Human Research Ethics Committee (HREC) Low Risk Executive Committee, University of Sydney, Protocol No. 14458).

### Results

**Figure 3:**
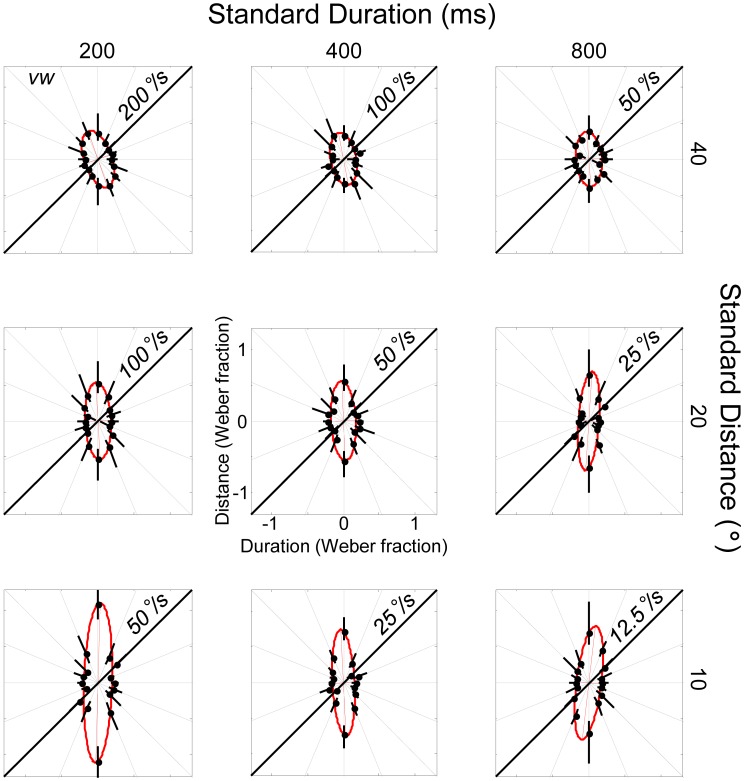
Motion discrimination contours for a single naïve observer for the 9 standards investigated in Experiment 1. The results for each individual standard value follow the conventions defined in Figure 1. Error bars for each threshold were obtained using a bootstrapping technique and correspond to 95% CIs. Ellipses were fit according to a non-linear least-squares technique.

Figure 3 shows the motion discrimination contours for a naïve observer (L1) who completed all nine standard conditions. The panels are arranged so that standard duration increases from left to right and standard distance increases from bottom to top; standard speeds are shown on the diagonal of each panel. Weber fractions within each panel follow the definitions given in Figure 1A.

**Figure 4:**
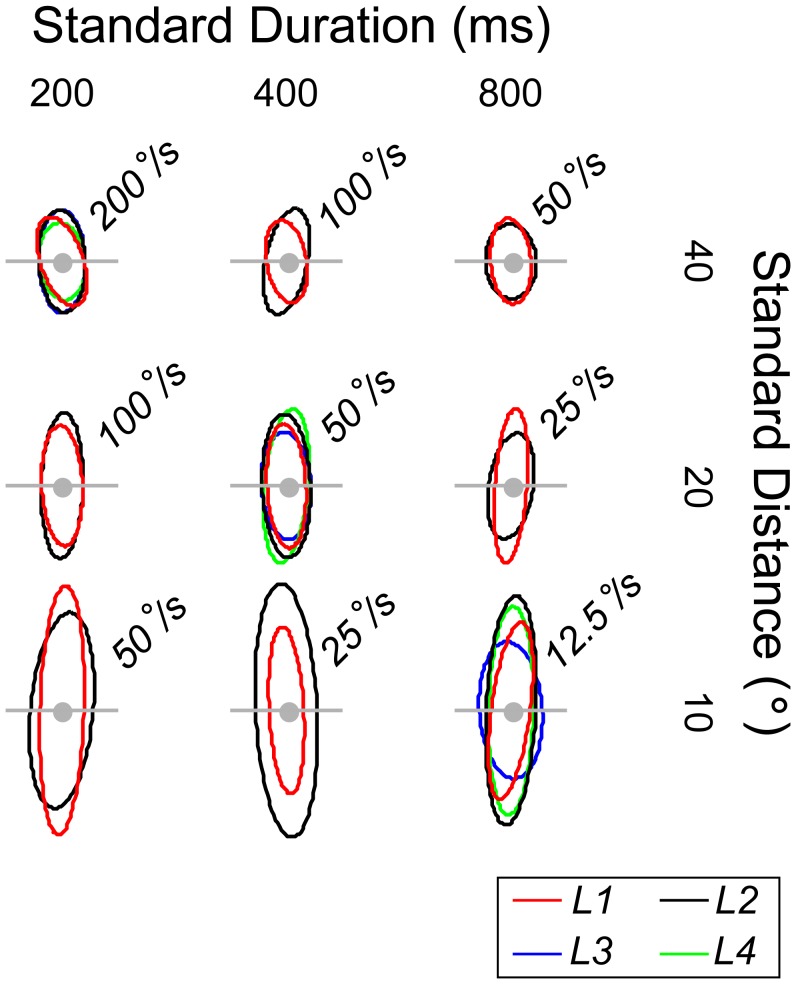
Summary of best-fitting ellipses across the four listeners (L1-L4) studied in Experiment 1. Two observers (L1, L2) completed all 9 conditions; two others (L3, L4) completed the 3 conditions lying on the major negative diagonal. The horizontal grey lines have length  = ±1 Weber fraction. All ellipses are oriented parallel to the axes of the distance-duration plane. Thus, one-sample t-tests for the mean ellipse orientations associated with the three standards on the major negative diagonal did not differ significantly from vertical (top-left: t(3) = 1.84, p>.10; middle: t(3) = .45, p>.50; bottom-right: t(3) = −0.81,p>.40). The results therefore provide no evidence that speed is used to discriminate test from standard; performance for all observers appears to be governed by separate estimates of distance and duration. The ellipses are stretched parallel to the Y axes, showing that duration discrimination was superior to distance discrimination.

All ellipses are oriented vertically rather than obliquely. The results therefore show that differences in speed did not determine performance for this listener. Figure 4 shows a summary of the ellipses obtained across the 4 listeners who took part in Experiment 1 (recall that L3 and L4 carried out a reduced set of conditions comprising the 3 standards along the major negative diagonal). As with listener L1, the ellipses are oriented close vertical (the results of one sample t-tests are given in the figure legend). Hence, there is little evidence that speed underlies performance for any of the listeners who took part in Experiment 1.

Since all ellipses are oriented vertically for each standard investigated, listeners appear to be more sensitive to changes in duration than distance. Moreover, the width of each ellipse appears to be independent of standard distance and speed, with the Weber fraction along the horizontal (ie. θ_i_ = 0) remaining roughly constant as standard duration increases. The latter finding suggests that duration discrimination obeys Weber's law over the range of standard durations tested. Conversely, distance discrimination does not appear to obey Weber's law because the height of each ellipse increases as standard distance declines. Thus, for the shortest distance investigated, distance discrimination is considerably worse in a proportional sense than at the two longer distances.

The observations about Weber's law assume that speed is not used by any of the listeners, an assumption that the overall orientation of the ellipses (and related statistics) seems to support. Distance and stimulus duration are therefore separable perceptual dimensions on the basis of these data. The fact that speed is not used when distance and duration cues are available could be interpreted in one of two ways: either the auditory system is unable to encode speed, or the encoding is largely ignored, perhaps because the underlying signals are noisy and so given less weight. Experiment 2 was designed to differentiate between these two alternatives by making distance and duration cues uninformative. If listeners are unable to encode speed, then they will be at chance for all θ. On the other hand, if they are able to encode speed at some point in the auditory system, the discrimination contours should rotate to become oriented obliquely along the iso-speed line.

## Experiment 2

**Figure 5:**
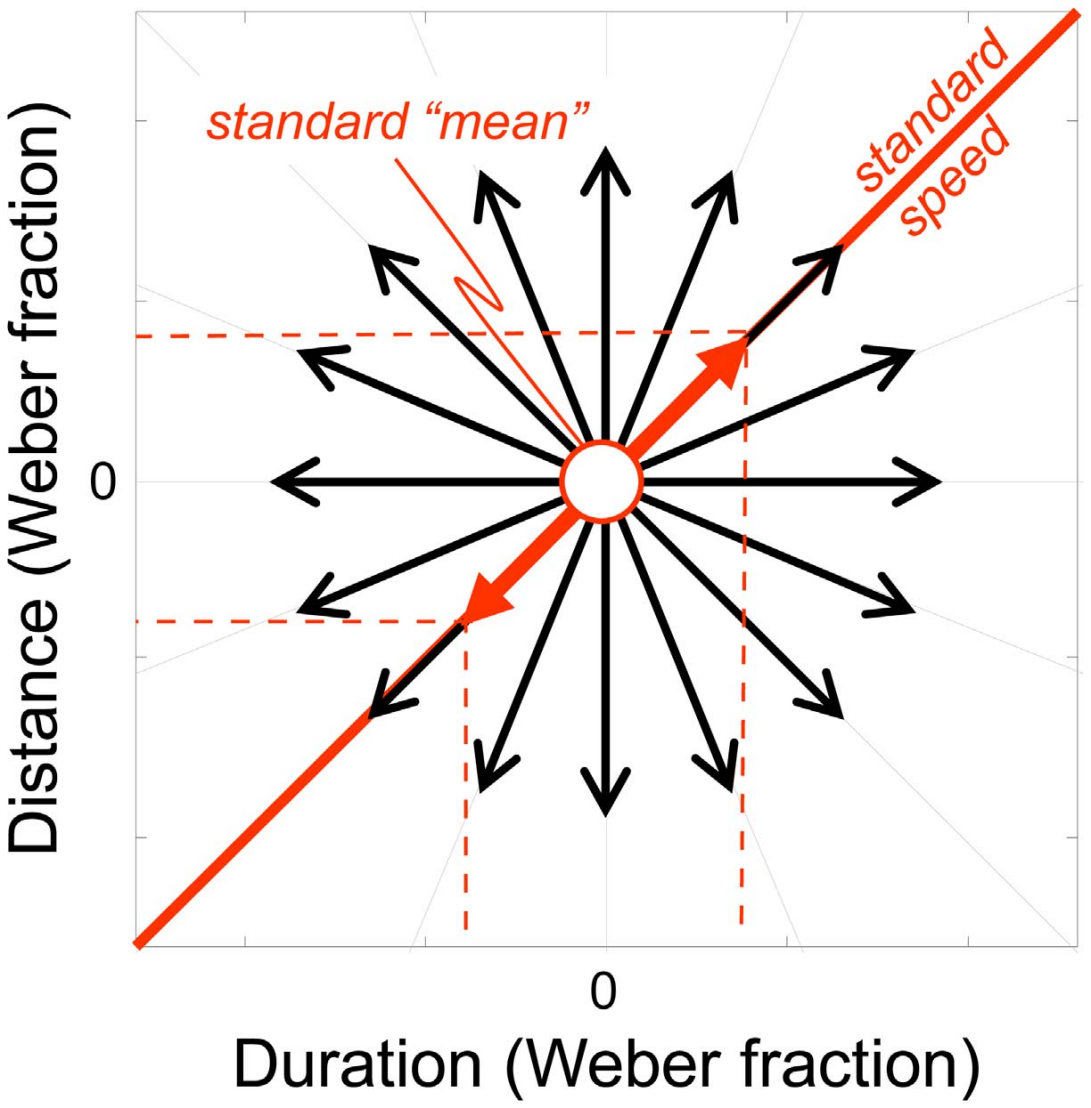
Isolating the speed cue. In Experiment 2, independent noise was added to the two standards to make distance and duration cues uninformative. This was achieved by defining a range of distances and durations from which to select the two standard stimuli, while ensuring that for each standard stimulus, the ratio of distance to duration (i.e. speed) was fixed and so constrained to lie on the 45° diagonal (thick red oblique arrow). Each standard selected in this manner was unique on each trial. The ranges used were defined individually for each observer. They were equal to 4 times the Weber fractions measured along the oblique (θ = 45°) in Experiment 1.

Experiment 2 used the manipulation suggested by Reisbeck & Gegenfurther (1999)[39], in which noise is added to the distance and duration components of the standard in such a way that their speed (i.e., their ratio) remains unchanged (see also Wardle & Alais (2013) [40]; note that Carlile and Best (2002) carried out a similar manipulation but for a two-interval task in which listeners knew to discriminate speed). This is shown graphically in Figure 5. On each trial, a standard duration is randomly selected from a predescribed range (vertical dotted lines), with the corresponding distance constrained to produce the required standard speed. Each standard selected in this manner was therefore unique on each trial. Listeners using either distance or duration cues to make their judgements would therefore be at chance in discriminating test from standard, forcing them to use speed.

### Methods

The stimuli and procedure were identical to those used in Experiment 1. The same listeners participated, which allowed us to tailor the amount of noise added to the standard based on each individuals' sensitivity found in Experiment 1. Specifically, the range of distances and durations used were equal to W_x_ = ±r.sin(θ_i_) and W_t_ = ±r.cos(θ_i_), with θ_i_ = 45° and r set to twice the Weber fractions measured in this direction in Experiment 1. The value of r therefore corresponds to half the length of the thick oblique red line shown in Figure 5. Note that only two of the listeners (L2, L3) knew that distance and duration cues had been made uninformative.

Discrimination contours for three standard speeds were investigated (12.5, 50 and 200 °/s), based on mean standard distances and durations pairs of (10°, 800 ms), (20°, 400 ms) and (40°, 200 ms). These corresponded to the standards lying along the negative obliques of Figures 3 and 4.

### Results

**Figure 6:**
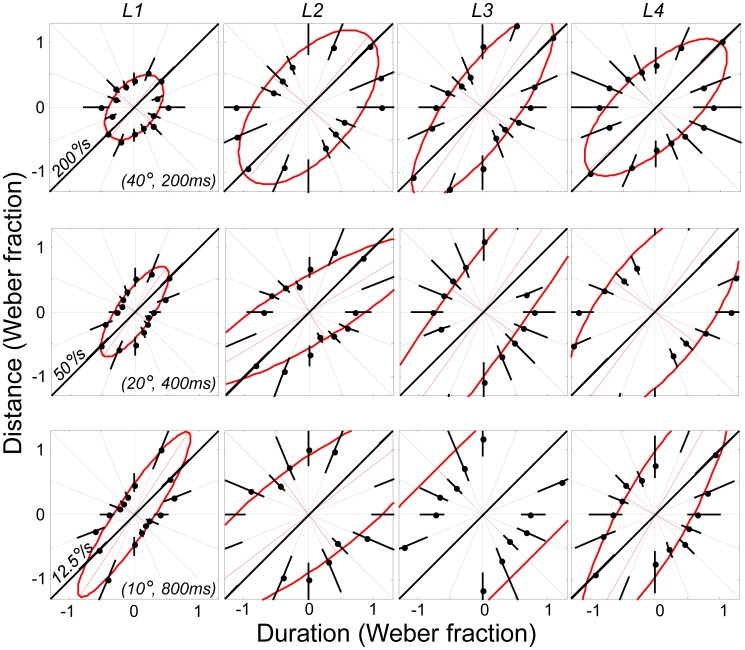
Results of Experiment 2, in which distance and duration noise were added to the standards to force discrimination based on speed. Each column corresponds to a different listener (L1-L4); each row is a different standard “mean”, corresponding to the standard values given along the major negative diagonal of Figures 3 and 4. The results show that the auditory system is sensitive to speed: when distance and duration cues are made uninformative, listeners are able to discriminate stimuli based on speed alone.

Figure 6 shows the results for all four listeners (columns) and all three standards (rows). With the addition of noise, the motion discrimination ellipses rotated from vertical to oblique, lying parallel to the iso-speed line. The results therefore show that listeners are able to use speed when forced to do so by making duration and distance cues unreliable. However, it is also the case that the thresholds are overall higher than in Experiment 1; in particular, the ellipses are wider along their minor diagonals, suggesting that performance was worse when listeners only have speed cues available to make their judgement.

**Figure 7:**
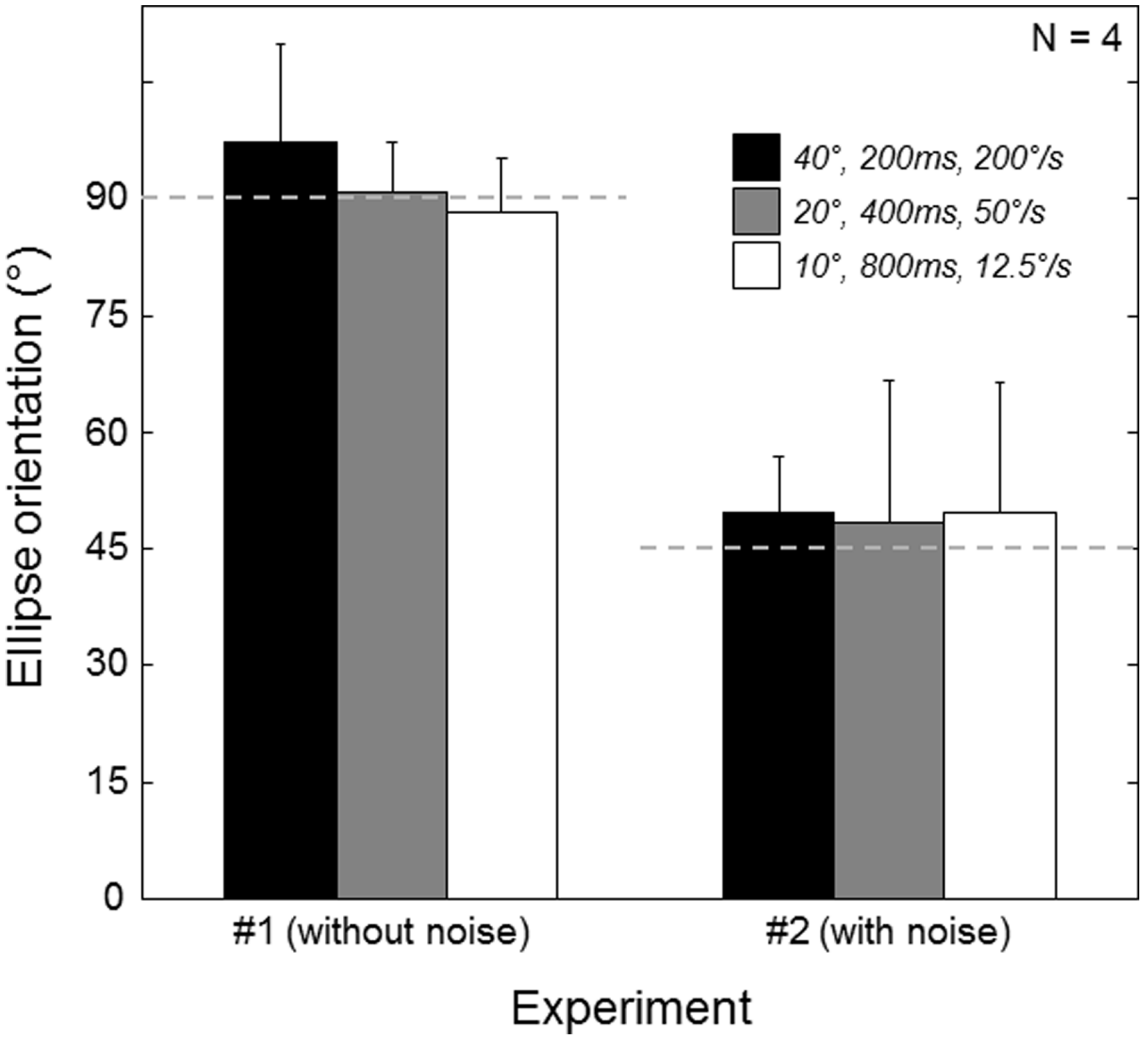
Comparison of Experiment 1 (without noise) and Experiment 2 (with noise) for the three common standards investigated (see legend). Without noise, the mean orientation of the major axis of the three ellipses was close to vertical (orientation = 90°). When speed noise was added, the mean was close to the iso-speed line oriented at 45°. Error bars correspond to 95% CIs.

Figure 7 summarises the findings of the two experiments, based on the three standards common to both. Each bar depicts the mean orientation of the ellipse across the four listeners, with the error bars showing 95% confidence intervals. For Experiment 1 (left hand bars), the means did not significantly differ from vertical (defined as 90°), showing the dominance of duration and distance cues over speed cues. When distance and duration cues were made uninformative in Experiment 2 (right-hand bars) the means did not significantly differ from the iso-speed line (defined as 45°), indicating that listeners were able to use speed cues when forced.

### Discussion

Motion discrimination thresholds were measured within the distance–duration plane in order to determine whether a combination of speed or distance and durations cue, or speed *per se*, dominates performance. If speed is the primary cue, then motion discrimination along lines of constant speed should be difficult and subsequent threshold contours elongated in this direction. Borrowing from colour vision (eg. [46]), these stimuli would form ‘metamers’ that cannot be differentiated near threshold, despite the fact that they are made from different combination of distance and duration. Finding evidence of metamers would then imply the existence of specialised motion detectors. Conversely, if speed is not the primary dimension limiting performance, and distance and duration are separable, then subsequent discrimination contours will align with the distance and duration axes. The results of Experiment 1 followed this pattern over a wide range of standard distances, durations and speeds: the discrimination contours were oriented vertically, implying better sensitivity to duration. However, when noise was added to the distance and duration cues as in Experiment 2, listeners were able to make use of speed information though performance was worse. Our data do not therefore support the idea that motion is precisely encoded early within the auditory system. Of course, we cannot rule out the possibility that low-level auditory motion detectors do exist but are overlooked by listeners when informative distance and duration cues are also made available. However, this does not seem a parsimonious explanation of our findings and so is not a view that we favour. Moreover, this view would have difficulty in explaining the domination of speed in visual discrimination tasks similar to the one used here [39], [47], given that in vision there is overwhelming evidence that such low-level motion detectors exist. If the auditory system contained similar motion mechanisms, we might expect a similar domination of speed.

The sounds used in the current experiments were made to move in an arc centred on the head, as is common in work on auditory motion [48]. One of the reasons for using circular trajectories is because a significant proportion of the motion created within the acoustic image corresponds to rotating the head in front of a largely stationary scene. However, by using horizontal motion, we effectively limited the motion information to dynamic changes in binaural cues (ILDs and ITDs), with spectral information primarily present to externalise the simulated sound sources. Of course, self-motion can cause the head to translate, as do some sources, and translation gives rise to additional cues to motion, such as monaural changes in frequency (the Doppler shift) and intensity. It is possible, therefore, that the study of different motion trajectories designed to include these additional motion cues may reveal specialised motion processing mechanisms not unveiled by our stimuli. In support of this possibility, Lutfi & Wang (1997)[49] and Kaczmarek (2005)[50] found that listeners give most weight to Doppler shifts when other motion cues are also present. Conversely, Neelon and Jenison (2003)[25] found no significant difference in the magnitude of auditory motion after effects when rotating and translating sources were compared.

Our findings should not be taken to mean that the speed of a moving sound is therefore an irrelevant dimension for the auditory system. For instance, Wuerger, Meyer, Hofbauer, Zetzsche & Schill (2010)[51] found that participants are able to judge the time-to-impact of auditory and visual stimuli with the same degree of precision and accuracy, once discriminability is equated. They went on to show that the precision of audio-visual time-to-impact judgements could be predicted from the precision of auditory and visual events when presented alone, suggesting optimal combination of motion information across these two modalities. Thus there appears to be some metric representation of speed information within the auditory system, though it does not seem to be encoded directly from the acoustic image.
